# Genetic Study of Total Phenolic Content and Antioxidant Activity Traits in Tetraploid Wheat via Genome-Wide Association Mapping

**DOI:** 10.3390/antiox14091048

**Published:** 2025-08-25

**Authors:** Ilaria Marcotuli, Francesca Vurro, Antonia Mores, Antonella Pasqualone, Pasqualina Colasuonno, Patricia Cabas-Lühmann, Andrés R. Schwember, Agata Gadaleta

**Affiliations:** 1 Department of Soil, Plant and Food Sciences, University of Bari Aldo Moro, 70126 Bari, Italy; francesca.vurro@uniba.it (F.V.); antonia.mores@libero.it (A.M.); antonella.pasqualone@uniba.it (A.P.); pasqualina.colasuonno@uniba.it (P.C.); agata.gadaleta@uniba.it (A.G.); 2 Departamento de Ciencias Vegetales, Facultad de Agronomía y Sistemas Naturales, Pontificia Universidad Católica de Chile, Santiago 306-22, Chile; pattycabas@gmail.com (P.C.-L.); aschwember@uc.cl (A.R.S.)

**Keywords:** tetraploid wheat, phenolic compound, antioxidant activity, QTL, candidate genes

## Abstract

Phenolic compounds contribute significantly to the nutritional and functional properties of wheat, particularly due to their antioxidant activity. In this study, a genome-wide association study was conducted to elucidate the genetic basis of total phenolic content (TPC) and antioxidant activity (AA) in a panel of 144 tetraploid wheat accessions representing diverse subspecies. The panel was evaluated under two different environments, located in Chile and Italy, to assess the influence of genotype, environment, and their interaction. Significant variability was observed for both TPC and AA, with TPC ranging from 0.26 to 0.82 mg gallic acid equivalent (GAE)/g and AA from 0.04 to 0.99 µmol Trolox equivalent (TE)/g. Substantial phenotypic variation and high broad-sense heritability were observed for both traits, underscoring the predominant genetic control. The genome-wide association study, using a mixed linear model (MLM), and the Bayesian information and Linkage-disequilibrium Iteratively Nested Keyway (BLINK) approaches identified 17 significant marker–trait associations, including quantitative trait loci on chromosomes 2B, 3A, 4B, 5A, 5B, and 6B. Notably, QTLs on chromosome 5A were co-localized for both TPC and AA, suggesting potential pleiotropic loci. Candidate genes linked to these loci included flavonol 3-sulfotransferase and peptidylprolyl isomerase, which are involved in phenylpropanoid metabolism and oxidative stress response, respectively. These findings offer valuable insights into the genetic basis of wheat phenolic traits and provide molecular targets for the development of biofortified cultivars through marker-assisted selection.

## 1. Introduction

In recent years, there has been a growing interest in healthier and more natural nutrition, particularly in functional foods, i.e., those food products whose value is based not only on their nutritional content, but also on their beneficial properties for health. This trend has driven scientific research to explore new food sources which are rich in bioactive compounds, i.e., substances capable of promoting health and preventing the onset of diseases. Among these, phenolic compounds play a key role due to their high antioxidant activity [1].

Wheat (*Triticum* spp.), in its bread (*Triticum aestivum* L. subsp. *aestivum*) and durum (*Triticum turgidum* L. subsp. *durum*) varieties, is one of the most widely cultivated and consumed crops worldwide. Its importance lies not only in its use in making bread, cookies, couscous, and pasta, but also in its nutritional value. In addition to providing carbohydrates, proteins, and vitamins, wheat is a valuable source of phenolic compounds, carotenoids, and flavonoids [2,3,4]. Local wheat landraces and traditional varieties represent a valuable reservoir of genetic diversity that remains underexploited in modern breeding programs. These genotypes, often adapted to specific agro-ecological conditions, can harbor unique alleles associated not only with nutritional traits, such as phenolic compound content, but also with key agronomic parameters, including yield stability, stress tolerance, and adaptation to marginal environments. Recent studies, such as the work by Tekin et al. [5], have highlighted the relevance of local germplasms in broadening the genetic base of wheat and in improving both compositional quality and productivity. Therefore, the investigation of local forms is essential for the development of more resilient and nutritionally rich wheat cultivars.

Interest in wheat phenolic compounds has increased due to their potential uses in reducing the risk of chronic diseases such as cardiovascular disorders, obesity, type 2 diabetes, and cancer [6,7]. Phenolic compounds are secondary metabolites of plants and play a fundamental role in plant growth, development, and defense, as well as contributing to their organoleptic properties such as color, taste, and aroma. Structurally, phenolic compounds are characterized by the presence of one or more phenolic groups (aromatic rings with hydroxyl groups) [8] and include a wide range of molecules such as phenolic acids, flavonoids, proanthocyanidins, stilbenes, and coumarins [9,10]. In wheat grains, the most abundant forms are phenolic acids and flavonoids [11], the greatest concentrations of which are mainly found in the outer layers of the grain, such as the bran and the aleurone layer [2,12].

However, variety, cultivation, and processing methods influence the concentrations and profiles of these phenolic compounds, causing, in many cases, a significant loss of beneficial substances [13,14]. This has led to renewed interest in the consumption of whole grains, which are richer in phenolic compounds in free, bound soluble, and insoluble forms, and in the study of their bioavailability and bioaccessibility.

The overall antioxidant activity (AA) of wheat products is considered to be an important indicator of nutritional quality and is commonly measured through spectrophotometric assays (e.g., 2,2′-azino-bis (3-ethylbenzothiazoline-6-sulfonic acid)—ABTS; 2,2-diphenyl-1-picrylhydrazyl—DPPH), which quantify the ability of metabolites to neutralize free radicals [15]. Several studies have highlighted that the levels of antioxidants and phenolics in wheat are strongly influenced by genetic background, but also by environmental factors and by the genotype × environment interaction [16].

Despite the importance of these traits, the genetic basis underlying phenolic compounds accumulation and AA in wheat remains partly unexplored, limiting the effectiveness of targeted breeding strategies. Furthermore, their genetic variability and heritability remain poorly characterized in large and genetically diverse populations. In this context, quantitative trait loci (QTLs) analysis represents an effective tool to identify genomic regions associated with complex traits, such as total phenolic content (TPC) and AA, and to identify candidate genes that could be useful for marker-assisted selection (MAS) [17]. Several studies have already identified QTL for nutraceutical compounds in durum and common wheats [18,19], but the integration of this information with environmental and functional data is still lacking. Recent mapping efforts have begun to elucidate the genetic architecture of key phenolic compounds in wheat. For instance, Shawai et al. [20] identified three stable QTLs (QFAC.caas-2D, QFAC.caas-3B, QFAC.caas-4D) associated with grain ferulic acid concentration in a Zhongmai 578 × Jimai 22 RIL population and converted them into functional KASP markers suitable for breeding programs. Furthermore, a GWAS in tetraploid wheat recently uncovered 22 QTLs linked to multiple phenolic acids, including FA and p-coumaric acid, with two QTLs co-localized at genes encoding *PAL2 and p-coumarate 3-hydroxylase* (*C3H*). In parallel, Zhi et al. [21] characterized QTLs for alkylresorcinol content in wheat and developed KASP markers to assist selection for health-promoting alkylresorcinols profiles.

The present study aims to address this knowledge gap by evaluating the variation in TPC and AA in a genetically diverse collection of tetraploid wheat accessions, of particular interest due to their rich genetic diversity and relevance for both breeding and nutritional studies. To capture the environmental influence on these traits and assess their stability, the accessions were cultivated under two highly contrasting field conditions—one in Chile and one in Italy—representing distinct agro-climatic zones. This dual-environmental approach enhances the reliability of trait detection and allows for the investigation of genotype-by-environment interactions. To further elucidate the genetic architecture underlying these traits, genome-wide association mapping (GWAS) was employed as a high-resolution approach to detect marker–trait associations across the genome.

GWAS leverages natural allelic diversity and historical recombination events within diverse germplasm panels, enabling the identification of QTLs and candidate genes associated with complex phenotypic traits, such as TPC and AA. The integration of genotypic and phenotypic data in this context provides a robust framework for uncovering genomic regions of interest and for developing molecular markers applicable in MAS.

The outcomes of this study are expected to enhance our understanding of the genetic basis of nutritionally relevant traits in wheat and support breeding strategies aimed at improving the health-promoting properties of wheat-based products.

## 2. Materials and Methods

### 2.1. Plant Material and Field Trial

A total of 144 accessions of tetraploid wheat (USDA gene bank collection) (*Triticum turgidum* L., 2n = 4x = 28; AABB genome) representing four subspecies (*dicoccum* (125 accessions), *paleocolchicum* (2), *polonicum* (8), and *turanicum* (9), previously described in Marcotuli et al. [22]) were used in this study. Field trials were conducted in two environments over two growing seasons: Valenzano, Southern Italy, during 2022, and Pirque, Central Chile, during 2021.

In Italy, the experiments were conducted at the ‘P. Martucci’ experimental station of the Department of Soil, Plant, and Food Sciences, University of Bari Aldo Moro (Italy), sited in Valenzano (Bari) (41°01′13.1″ N, 16°54′12.9″ W). The plant material was sowed from 22 to 26 November 2021 and harvested from 4 to 8 of July. The temperature, rainfall, humidity, and other meteorological data for the season are reported in the Supplementary Materials Table S1. The soils at the ‘P. Martucci’ Experimental Station are medium–fine-textured and lie over cretaceous limestone. Due to deep tillage and rock fragmentation, soil composition varies across the field. Undisturbed areas contain mostly fine earth, while cultivated plots are rich in skeletal material—comprising up to 76% in the topsoil. Rock fragments are mainly of medium size, and disturbed soils show higher calcium carbonate levels and pH, but lower organic carbon and nitrogen compared to natural soils. In Chile, trials were performed at the Pontifical Catholic University experimental station in Pirque (33°40′00″ S, 70°35′00″ W) during the 2021 growing season (August–December); sowing took place from 2 to 6 August 2021 and harvest was conducted from 17 to 21 January 2022. The meteorological conditions of the Pirque experimental station during the growing season are reported in the Supplementary Materials Table S2. The experimental field soil in Pirque is classified as a sandy loam, formed by alluvial sediments and characterized by medium fertility. It has a slightly alkaline pH of 7.89 (in water) and moderate organic matter content (3.15%). Nutrient analysis shows good availability of nitrogen, phosphorus, and potassium. The soil’s cation exchange capacity demonstrates that calcium is the dominant base. The micronutrient levels are adequate, with notable values for iron, manganese, and zinc, indicating a balanced nutrient profile suitable for crop growth.

A randomized complete block design (RCBD) with three replications was employed across all environments. Each plot consisted of one 1 m row, and the plots were spaced 30 cm apart, sown with 80 viable seeds.

In Italy, basal fertilization included 68 kg N/ha and 46 kg P_2_O_5_/ha, applied before sowing. An additional 32 kg N/ha was supplied at the stem-elongation stage. Irrigation was provided only in the absence of rainfall during the critical developmental stages and was discontinued once the kernels reached the waxy stage. All plots were harvested manually when the ears were dry (12–14% moisture).

In Chile, basal fertilization consisted of 51 kg N/ha, 69 kg P_2_O_5_/ha, and 53 kg K_2_O/ha. An additional 184 kg N/ha was applied during tillering. Irrigation was managed to prevent drought stress throughout the growth cycle, and weeds were controlled chemically before and after sowing. Plots were hand-harvested and threshed with about 14% moisture content.

### 2.2. TPC and AA in Wheat Grains

The extraction and quantification of TPC was carried out as described by Pasqualone et al. [23], with minimal modifications. Briefly, 1 g of sample was extracted with 5 mL of methanol/water at 80:20 *v/v* in an ultrasonic bath (CEIA international S.A., 115/230 Vac 1- 50/60 Hz–400 VA max, Viciomaggio, Italy) for 15 min at room temperature; then, it was shaken for 30 min and centrifuged (Thermo Fisher Scientific, Osterode am Harz, Germany) for 10 min at 12,000× *g* at 4 °C. Two independent extractions were performed per sample as analytical replicates. Then, 100 µL of the filtered extract was mixed with 900 µL of deionized water and 100 µL of Folin–Ciocalteu reagent. After 3 min, 800 µL of a 7.5% (*w/v*) water solution of Na_2_CO_3_ was added, followed by 60 min incubation in the dark. The samples were then centrifuged (Biofuge Pico microcentrifuge, Heraeus instrument, Hanau, Germany) for 3 min at 7800× *g*. The spectrophotometric quantification was carried out at 720 nm, using a Cary 60 UV–Vis spectrophotometer (Agilent Technologies, Santa Clara, CA, USA). The results were expressed as mg gallic acid equivalent (GAE)/g of the sample.

For the determination of AA, the same extracts were submitted to a radical scavenging assay using 1,1-diphenyl-2-picrylhydrazyl (DPPH) radical, according to Pasqualone et al. [23], with minor modifications. A measure of 50 µL of the extract was added to 950 µL of a 0.08 mM DPPH ethanol solution. After 30 min of incubation in the dark, the spectrophotometric absorbance was read at 517 nm (Agilent Technologies, Santa Clara, CA, USA). The results were expressed as µmol Trolox equivalent (TE)/g of the sample. For each extract, three separate aliquots were submitted to spectrophotometric determinations as technical replicates.

### 2.3. Statistical Analysis

All the collected data of the 144 genotypes were subjected to analysis of variance (ANOVA) at a 95% confidence level (F tests: *p* ≤ 0.05) using the Rstudio^®^ variability package. Broad-sense heritability (*H*^2^) was estimated as the proportion of genetic variance (σ^2^g) to phenotypic variance using the Rstudio^®^ package lme4. Heritability values were categorized as follows: <0.5 indicates low, 0.5 to 0.75 indicates moderate, 0.75 to 0.9 indicates high, and >0.9 indicates very high broad-sense heritability.

### 2.4. QTL and Candidate Gene Detection

Genotyping of the wheat collection was performed using the 7K iSelect SNP array developed by Illumina CSPro^®^ (San Diego, CA, USA), targeting 6731 single-nucleotide polymorphisms (SNPs). Genomic DNA (1 µg per sample) was extracted and genotyped at TraitGenetics GmbH (Gatersleben, Germany) following the protocol described by Akhunov et al. SNP hybridization and detection were carried out using the Illumina iScan platform, and genotype calling was performed using GenomeStudio software v2011.1 (Illumina CSPro^®^, Illumina, San Diego, CA 92122, USA).

Quality control filtering was applied prior to the genome-wide association analysis. Markers with a minor allele frequency (MAF) < 10% and those with >5% missing data were excluded using GenAlEx software version 6.5 (The Australian National University, Canberra, Australia) resulting in a curated dataset of 3942 high-quality SNPs.

Genome-wide association studies (GWASs) were performed using the Genomic Association and Prediction Integrated Tool (GAPIT v3) [24] in R, employing two statistical models: the mixed linear model (MLM) [25] and Bayesian-information and Linkage-disequilibrium Iteratively Nested Keyway (BLINK) [26]. Both models accounted for population structure and relatedness through the inclusion of the Q matrix and the kinship matrix (K). Marker–trait associations were considered significant at −Log10(*p*) > 3, and for each significant SNP, the *R*^2^ (explained variance) and marker effect were recorded.

To identify putative candidate genes within QTL regions, SNP sequences associated with significant trait loci were used as queries in BLAST (https://blast.ncbi.nlm.nih.gov/Blast.cgi?PROGRAM=blastn&PAGE_TYPE=BlastSearch&LINK_LOC=blasthome, accessed on 20 March 2025) analyses against the SVEVO genome via the GrainGenes database (https://graingenes.org/GG3/, accessed on 20 March 2025) and the CerealDB SNP repository (https://www.cerealsdb.uk.net, accessed on 20 March 2025). Genes with the highest sequence homology in relevant biosynthetic pathways were selected as candidates for functional annotation and further investigation.

## 3. Results

The genetic control of TPC in wheat (*Triticum* spp.) is quantitative and polygenic; therefore, environmental influences and agronomic management can modify the expression of genes involved in phenolic biosynthesis. To investigate the genetic basis of TPC and AA, phenotypic evaluations were conducted across multiple environments, and genome-wide association studies (GWASs) were performed.

### 3.1. TPC and AA in Tetraploid Wheats

To evaluate the natural variation in nutraceutical traits, TPC and AA were measured in 144 tetraploid wheat accessions grown under field conditions in Chile and Italy. These parameters, assessed using spectrophotometric assays, are summarized in Table 1, which presents a subset of phenotypic data across replicates, confirming the reproducibility of measurements and illustrating the range of expression among accessions. Data were collected from two biological replicates per genotypes, and results are expressed as gallic acid equivalents (mg GAE/g) for TPC and Trolox equivalents (µmol TE/g) for AA.

The TPC values ranged from 0.26 to 0.82 mg GAE/g across the two environments, with most samples clustering between 0.40 and 0.60 mg GAE/g. The phenolic content was quantified from the free phenolic fraction using 80% methanol, a solvent efficient at solubilizing low-molecular-weight phenolics. Bound phenolics, i.e., those complexed with polysaccharides or proteins, were not assessed in this study, though they also contribute to antioxidant capacity and bioactivity. The antioxidant activity also varied substantially among genotypes, with DPPH values ranging from 0.04 to 0.99 µmol TE/g.

Notably, samples with higher TPC values did not always show proportionally higher AA values, highlighting that phenolic composition, not just concentration, influences antioxidant capacity.

### 3.2. Heritability and Environmental Effects on Phenolic Traits and Candidate Gene Identification

Analysis of variance (ANOVA) revealed highly significant genotypic effects (*p* < 0.001) for both TPC and AA across environments (Table 2), confirming strong genetic control over these traits. A significant genotype × environment (G × E) interaction was also observed, indicating variable genotypic responses across the Italian and Chilean growing sites.

Despite the environmental influence on trait expression, heritability estimates were remarkably high in both environments. For TPC, broad-sense heritability (*H*^2^) was estimated at 0.72, while AA exhibited *H*^2^ values of 0.65. These results suggest that the phenotypic variance is predominantly attributable to genetic variation, with minimal environmental noise, making both traits suitable targets for breeding programs.

**Figure 1:**
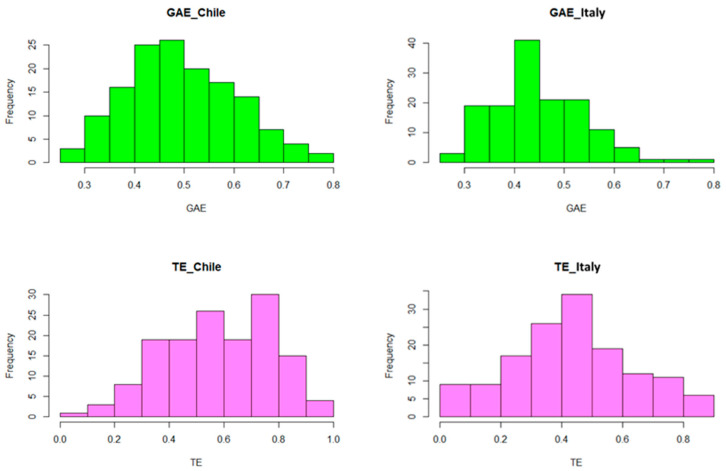
Frequency distribution of total phenolic compounds, expressed as gallic acid equivalent (GAE), and antioxidant activity, expressed as Trolox equivalent (TE), for 144 *Triticum turgidum* accessions grown in Chile and Italy.

Visual inspection of the frequency distribution (Figure 1) confirmed continuous phenotypic variation for both TPC and AA, consistent with polygenic inheritance. The Chilean environment tended to produce slightly higher average values and broader ranges, likely reflecting greater environmental heterogeneity or stress exposure (e.g., UV radiation, water limitation), which may have triggered phenylpropanoid biosynthesis.

Notably, genotype PI94682 (*T. turgidum* subsp. *dicoccon*) exemplified strong environmental responsiveness, with AA values ranging from 0.05 µmol TE/g in Italy to 0.83 µmol TE/g in Chile, highlighting the role of G × E interaction in trait modulation.

### 3.3. Genome-Wide Association Study and QTL Detection

To uncover the genetic architecture underlying TPC and AA, a GWAS was conducted using 3942 high-quality polymorphic SNPs after filtering for MAF (<10%) and missing data (>5%). Both the mixed linear model (MLM) and the BLINK algorithms implemented in GAPIT v3 were used to identify significant marker–trait associations.

A total of 17 significant marker–trait associations were identified at a significance threshold of −Log10(*p*) > 3, encompassing four QTLs for TPC and thirteen QTLs for AA (Table 3). Several loci were environment-specific and were detected by both models and/or in multiple environments. Owing to the substantial environmental differences between the two locations and the genetic variability inherent in the wild germplasm used for the analysis, no quantitative trait loci were consistently detected across both environments. Only a single QTL was identified in the Italian environment as well as in the across-environment mean analysis. This QTL appeared to be strongly influenced by a significant genotype-by-environment (G × E) interaction.

**Figure 2:**
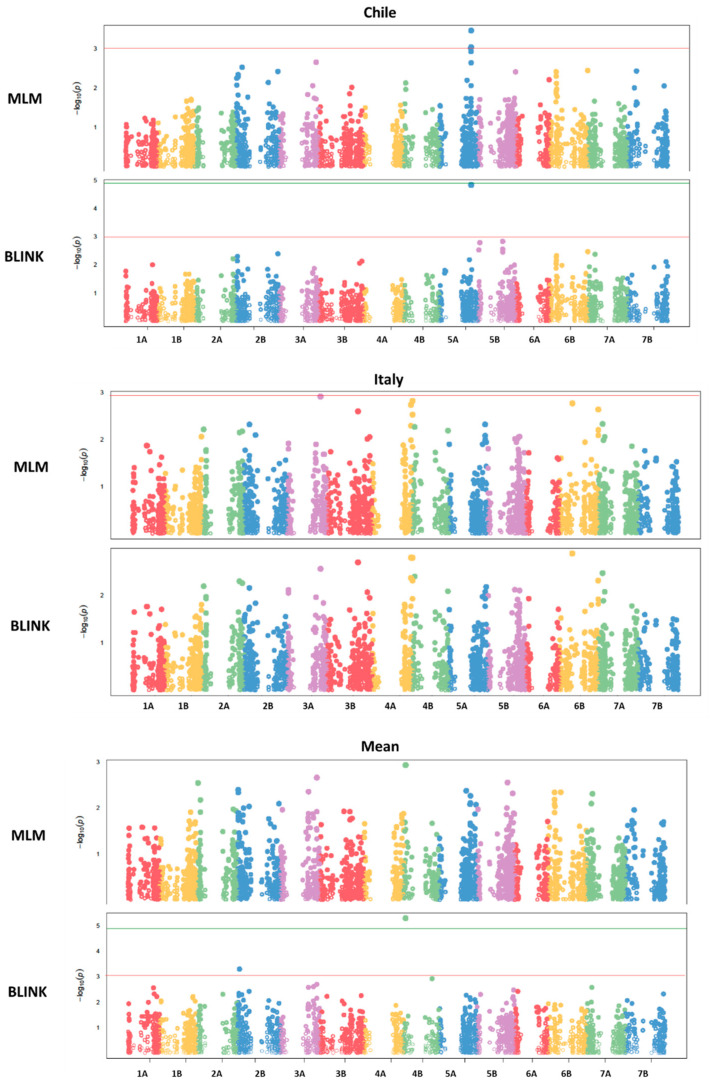
Manhattan plot of total phenolic compounds from GWAS using the mixed linear model and the BLINK model. The −log10 (*p*-values) from the GWAS are plotted according to the genetic position of the SNP markers on each of the 7 wheat chromosome pairs. In the figure, the green line indicates a threshold of −log10 (*p*-value) > 5, while the red line represents −log10 (*p*-value) > 3.

**Figure 3:**
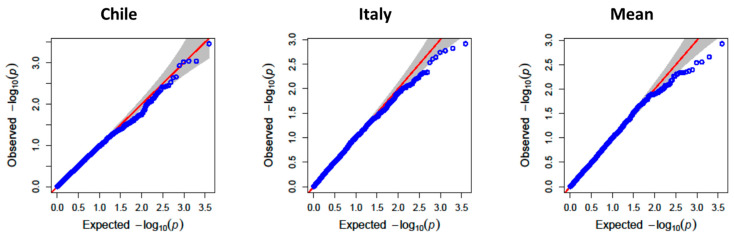
QQ-plot of observed against expected probability values (*p*-values) from the genome-wide association analysis of total phenolic compounds using the mixed linear model.

**Figure 4:**
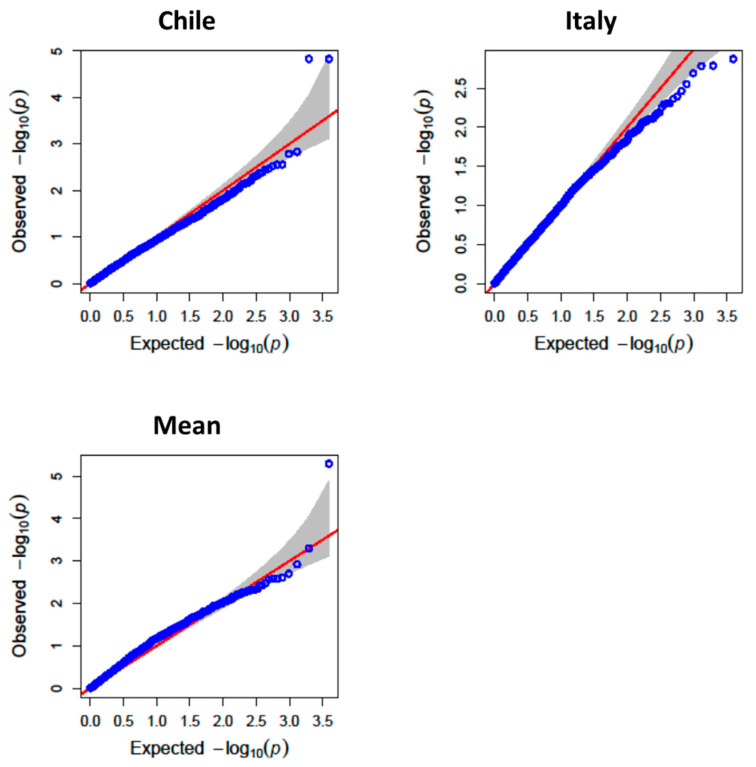
QQ-plot of observed against expected probability values (*p*-values) from the genome-wide association analysis of total phenolic compounds using the BLINK model.Among the candidate genes mapped in proximity to these QTLs, a flavonol 3-sulfotransferase gene (on 3A) and a peptidylprolyl isomerase gene (on 5B) were identified. The former is implicated in secondary metabolism, while the latter plays a role in stress response and protein folding under oxidative conditions, supporting their potential involvement in phenolic biosynthesis and antioxidant regulation.

For TPC, major QTLs were identified on chromosomes 2B, 3A, 4B, and 5A (Figure 2, Figure 3 and Figure 4). The QTL QGae.bc.5A, located at 581.5 Mb on chromosome 5A, was particularly notable, showing an LOD score of 4.83 and explaining 14% of the phenotypic variance. This locus co-localized with QTe.bc.5A-2, a QTL associated with AA, suggesting potential pleiotropic effect or tight linkage of the candidate genes. 

**Figure 5:**
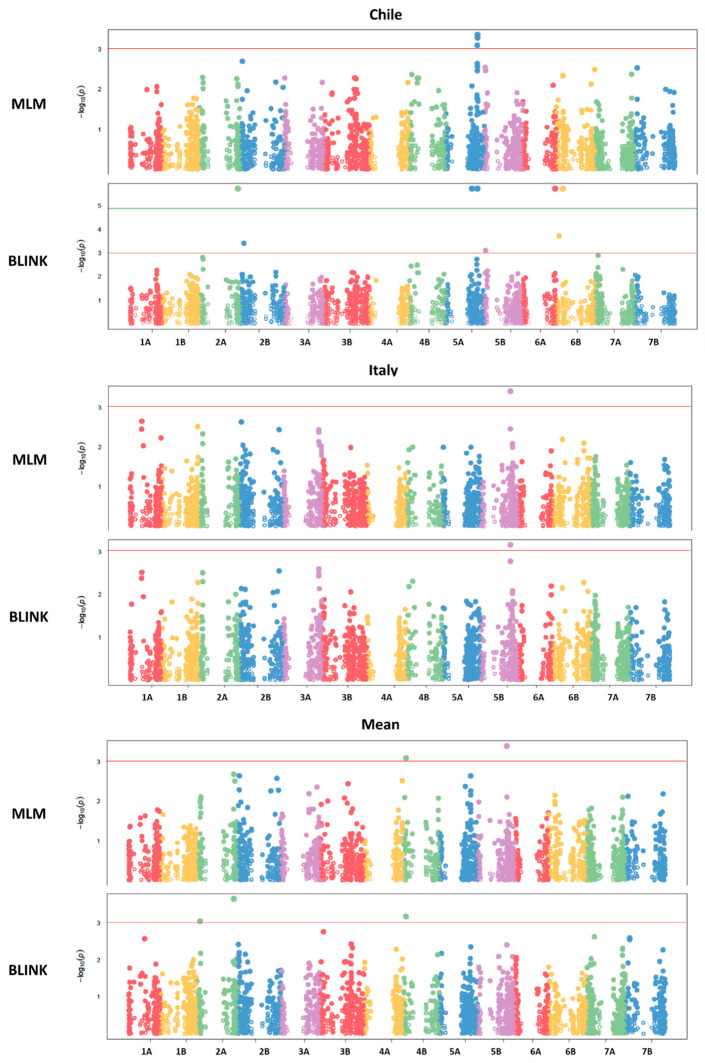
Manhattan plot of antioxidant activity from GWAS using the mixed linear model and the BLINK model. The −log10 (*p*-values) from the GWAS are plotted according to the genetic positions of the SNP markers on each of the 7 wheat chromosome pairs. In the figure, the green line indicates a threshold of −log10 (*p*-value) > 5, while the red line represents −log10 (*p*-value) > 3.

**Figure 6:**
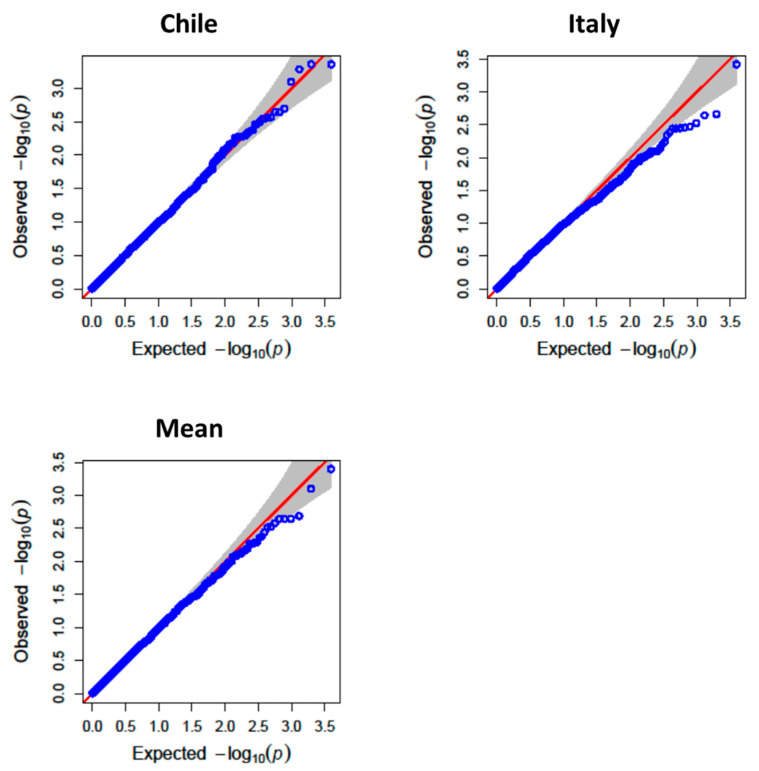
QQ-plot of observed against expected probability values (*p*-values) from the genome-wide association analysis of antioxidant activity using the mixed linear model.

**Figure 7:**
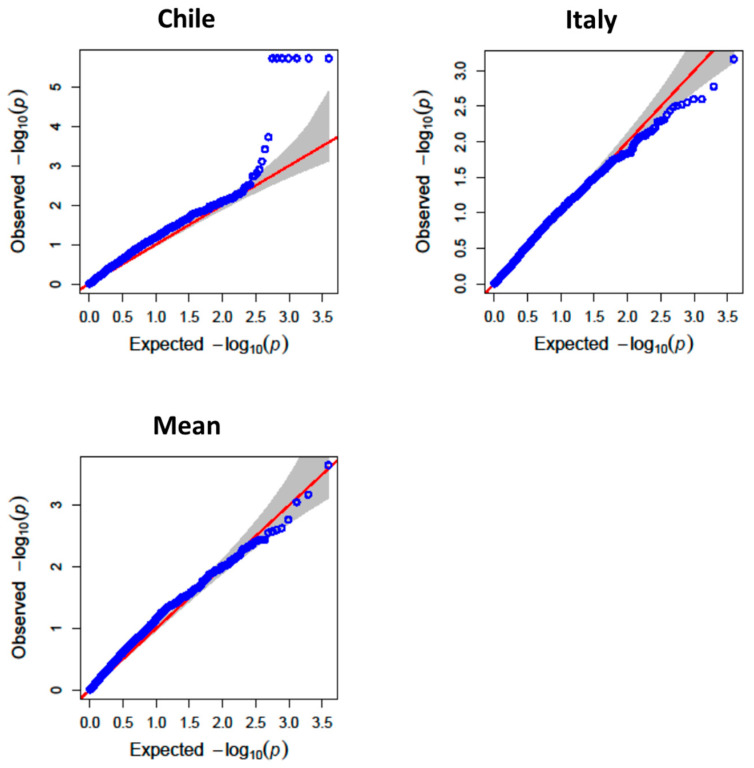
QQ-plot of observed against expected probability values (*p*-values) from the genome-wide association analysis of antioxidant activity using the BLINK model.

For AA, QTLs were distributed across multiple chromosomes, with notable associations on 2A, 2B, 4B, 5A, 5B, 6A, and 6B (Figure 5, Figure 6 and Figure 7). The most significant QTL, QTe.bc.5A-1, explained 17% of the phenotypic variance in the Chilean environment and had the highest LOD score (5.73). The co-localization of several QTLs for both traits further supports a partially shared genetic basis.

To refine candidate gene discovery, SNPs within significant QTL intervals were used in the BLAST analyses against the SVEVO reference genome and the CerealDB SNP database. Sequences showing high homology to genes involved in flavonoid modification and stress response were prioritized. The identification of genes such as flavonol 3-sulfotransferase and peptidylprolyl isomerase is consistent with previous findings on the functional roles of these proteins in phenylpropanoid metabolism and antioxidant mechanisms.

These loci and their associated markers represent valuable resources for marker-assisted selection and provide a foundation for further functional validation through gene expression or knockout studies.

As shown in Figure 1, the TPC of samples grown in Italy shows a narrower distribution with a clear peak, suggesting a less variable environment; meanwhile, in Chile, the greater dispersion and symmetry indicate that more heterogeneous environmental conditions could have influenced phenolic synthesis. The ANOVA (Table 2) shows that the environment has a highly significant effect on the TPC. In other words, the mean values are significantly different between the samples grown in Italy and those grown in Chile. This visually supports what was observed in the histograms, i.e., in Chile, a tendency to higher mean values and greater variability was observed, and in Italy, more concentrated and slightly lower values were observed.

The AA values show a moderate (Bari)–high (Chile) variability (Figure 1). The Chilean environment seems to favor a greater expression of antioxidant activity, probably due to environmental stresses (e.g., UV radiation or drought).

Phenotypic plasticity is evident; the same genotype can express very different levels of TE depending on the environment, suggesting a G × E interaction.

Also, for AA, the ANOVA shows that the environmental factor has a highly significant effect. This visually confirms what was observed from the histograms, i.e., the Chilean environment tends to generate higher AA values than the Italian one.

Table 2 summarizes the mean, standard deviation (SD), median, range, and broad-sense heritability (*H*^2^) for TPC and AA per environment (*n* = 144). Phenotypic data analysis revealed a significant difference in TPC between the two environments. On average, genotypes evaluated in Chile showed a higher value (0.50 ± 0.02 mg GAE/g) than those grown in Italy (0.45 ± 0.02 mg GAE/g). TPC showed a continuous distribution and a significant inter-genotypic variability in both environments, with values ranging from 0.27 to 0.82 mg GAE/g in Chile and from 0.26 to 0.79 mg GAE/g in Bari.

The *H*^2^ estimate for phenolic content was high (*H*^2^ = 0.72), indicating a strong genetic component of the trait.

Similarly, AA showed higher mean values in Chile (0.58 ± 0.01 µmol TE/g) than in Italy (0.44 ± 0.01 µmol TE/g). The minimum and maximum values were 0.08–0.99 µmol TE/g in Chile and 0.04–0.89 µmol TE/g in Italy. Also, in this case, the phenotypic distribution was broad and continuous.

The broad-sense heritability for AA reached a value of *H*^2^ = 0.65, indicating that, even for this trait, the observed variation is attributable to genetic factors.

## 4. Discussion

Overall, the high inter-genotypic variability and consistency between the replicates supports the presence of significant genetic diversity for both TPC and AA in the wheat panel. The observed values of TPC were consistent with those previously reported in durum wheat by Kosma et al. [27], Bellato et al. [28], and Martini et al. [29], though slight differences may reflect variations in genotype, environment, or extraction method. The observed values of AA, instead, were generally lower than those reported in earlier studies using similar assays [30,31], possibly due to differences in extraction protocols, wheat species, or environmental stressors.

The contribution of phenolic compounds to antioxidant properties in wheat is well documented, with high phenolic intake being associated with reduced risk of chronic diseases such as cardiovascular disease, type 2 diabetes, and certain cancers. The TPC and AA values found were not particularly high, but wheat is the basis of foods consumed daily; therefore, even if they are only present in low amounts, the bioactive elements a food contains can have a significant impact on health. Recent comparative studies on different wheat species, including einkorn, emmer, spelt, and pigmented genotypes, have revealed significant variation in phenolic content and antioxidant activity. Ancient and pigmented wheats generally show higher levels than conventional wheats [32,33]. Among these, pigmented genotypes with dark pericarp, specifically black and purple–blue hues, have been shown to have higher concentrations of phenolic compounds, including anthocyanins and phenolic acids, which may have positive health implications [33]. Notably, the localization of phenolic substances is known to be most concentrated in the peripheral layers of the caryopsis, which is one more reason to consume whole wheat foods or to recover wheat milling by-products in the formulation of functional foods [34]. However, the accumulation of these compounds, especially in bran layers, can lead to darker flour coloration due to enzymatic browning prompted by polyphenol oxidase (PPO), which may negatively impact the sensory appeal of end-products like pasta [35]. This trade-off between nutritional and technological quality requires breeding strategies that combine high phenolic content with low PPO activity. In this context, the genetic mapping of PPO activity provides a valuable tool for targeted selection. Simeone et al. [36] successfully mapped a major locus controlling PPO activity on the long arm of chromosome 2A in tetraploid wheat, identifying a strong association with the RFLP marker Xutv1427-2A. This molecular marker represents a promising resource for marker-assisted selection aimed at reducing PPO activity and, consequently, undesirable browning in wheat-based foods.

This study demonstrates that TPC and AA in tetraploid wheat are highly heritable, environmentally responsive, and genetically complex traits. The strong genetic control, confirmed by high heritability estimates across environments, suggests excellent prospects for improving these traits through breeding.

*H*^2^ values were 0.72 for TPC. This confirms that genetic variability among genotypes is the main determinant of the observed phenotypic differences, as already reported in the literature for secondary metabolites in bread and durum wheat [37,38,39].

The high heritability observed for AA (*H*^2^ = 0.65) is particularly relevant, as it suggests that genetic selection of genotypes with high AA can be extremely effective, with minimal environmental influence. Similar results were also reported by Liu et al. [16], who observed high levels of heritability for this trait in wheat populations [40].

Despite the higher averages found in Chile compared to Italy, the high *H*^2^ value for both traits suggests that environmental differences mainly influence the absolute level of the traits but not the ranking of the genotypes. This is consistent with what was observed by Dinelli et al. [41], according to which the TPC and AA in wheat are strongly influenced by the genotype, even if modulated by pedoclimatic conditions [42]. This means that a genotype showing high performance in one environment is highly likely to maintain good performance also in another environment, despite any variations in the absolute values of the trait. This stability in the “ranking” of genotypes is particularly advantageous in breeding programs, since it allows reliable selection even in environments different from the target ones. The evaluation of the wheat panel in two contrasting environments (Chile and Italy) offered an important opportunity to explore the environmental plasticity and stability of phenolic content and antioxidant activity across diverse agro-ecological conditions. These two environments differ significantly in terms of climate (e.g., temperature, rainfall), altitude, soil characteristics, and growing season length, all of which can influence the accumulation of secondary metabolites in wheat grains. In Valenzano (Italy), the field trials were conducted on medium–fine-textured soils formed over Cretaceous limestone, with high skeletal content and elevated calcium carbonate levels due to intensive rock fragmentation. These soils are characterized by higher pH and lower organic carbon and nitrogen levels, potentially affecting nutrient availability and plant metabolic responses [43]. Conversely, in Pirque (Chile), the trials took place on a sandy loam soil derived from alluvial sediments, with medium fertility, a balanced micronutrient profile, and slightly alkaline pH—conditions generally more favorable for root development and nutrient uptake.

Although the results highlighted significant genotypic differences and high heritability estimates for both TPC and AA, suggesting strong genetic control, the environmental component remains relevant. The consistently higher average values recorded in Chile compared to Italy point to an effect of environment on the absolute expression of the traits, likely linked to environmental stressors or differences in plant development stages at harvest. Despite this, the ranking of genotypes remained relatively stable across locations, indicating that, while the magnitude of expression is environment-dependent, the relative performance of genotypes is preserved; this is a key aspect for selection in breeding programs.

This pattern is consistent with the presence of genotype × environment (G×E) interactions, which, although not the primary focus of this study, merit further exploration. G×E interactions can obscure or enhance genetic effects, depending on environmental context, and their proper evaluation is essential to identify genotypes with robust performance across diverse environments or those specifically adapted to certain conditions.

By confirming the genetic stability of key bioactive traits and acknowledging their environmental modulation, this study underscores the value of conducting phenotypic evaluations in geographically and climatically distinct sites. Such an approach enhances the reliability of genotype selection and increases the likelihood of developing resilient, health-promoting wheat varieties that are adaptable to future climate challenges. These results support the use of direct or MAS strategies to improve the TPC and AA in wheat. The strong influence of the genotype and the relative stability of the traits in different environments make it possible to identify elite genotypes adaptable to different environments for the development of cultivars with high nutraceutical value.

The results obtained by the GWAS highlight a complex and partially shared genetic basis between TPC and AA in wheat. The co-localization of QTL for both traits on chromosome 5A, in particular the marker BS00065481_51 (Table 4), suggests the presence of pleiotropic loci influencing both traits, consistent with the dual role of phenolic compounds as structural and antioxidant agents [18,44,45]. The identification of a candidate gene as *flavonol 3-sulfotransferase* further supports the involvement of secondary metabolism in the modulation of TPC and AA, since the sulfation of flavonoids can alter their stability and bioactivity [46]. Furthermore, the association of QTL *QTe.bc.5B-2* with a gene encoding a *peptidylprolyl isomerase* highlights a possible connection with oxidative stress response mechanisms, given the known role of these isomerases in proteostasis and stress tolerance [47].

In light of the relationship between phenolic compound accumulation and technological quality issues, such as enzymatic browning, future breeding efforts should aim to identify genotypes that combine high TPC and AA with reduced polyphenol oxidase (PPO) activity. PPO, primarily localized in the outer layers of the caryopsis, catalyzes the oxidation of phenolics leading to the formation of undesirable dark pigments in wheat-based products. This enzymatic browning can negatively affect consumer acceptance, particularly in products like pasta. In this regard, the findings of Simeone et al. [36] provide a useful resource: their genetic mapping study identified a major locus for PPO activity on the long arm of chromosome 2A in tetraploid wheat and reported a strong linkage with the RFLP marker Xutv1427-2A [48]. The availability of such molecular markers enables the simultaneous selection for reduced PPO activity alongside enhanced nutraceutical quality, offering a promising path toward developing wheat cultivars that are both healthy and appealing in terms of appearance and processing performance.

## 5. Conclusions

Overall, this study provides new insights into the genetic architecture of total phenolic content (TPC) and antioxidant activity (AA) in tetraploid wheat, highlighting their high heritability, environmental responsiveness, and partial genetic overlap. Genome-wide association mapping (GWAS) enabled the identification of key loci and candidate genes involved in secondary metabolism and oxidative stress response. The integration of these findings with previous knowledge on polyphenol oxidase (PPO) activity, particularly the genetic mapping of PPO on chromosome 2A, opens new perspectives for breeding strategies that aim to enhance the nutraceutical value of wheat while minimizing undesirable enzymatic browning. These results support the development of molecular tools for marker-assisted selection and the design of wheat cultivars with improved health benefits and technological quality.

## Figures and Tables

**Table 1:** Total phenolic compounds and antioxidant activity of samples grown in Pirque (Chile) in 2021 and in Valenzano (Italy) in 2022.

Samples	Species	Total Phenolic Compounds		Antioxidant Activity	
		mg GAE/g (d.m.)		µmol TE/g (d.m.)	
		Chile	Italy	Chile	Italy
PI387744	*Triticum turgidum* subsp. dicoccon	0.45 ± 0.02	0.44 ± 0.00	0.61 ± 0.02	0.63 ± 0.00
PI384263	*Triticum turgidum* subsp. *dicoccon*	0.59 ± 0.01	0.57 ± 0.00	0.74 ± 0.01	0.85 ± 0.01
PI362500	*Triticum turgidum* subsp. *dicoccon*	0.69 ± 0.02	0.72 ± 0.05	0.84 ± 0.00	0.88 ± 0.02
PI355471	*Triticum turgidum* subsp. *dicoccon*	0.80 ± 0.03	0.77 ± 0.03	0.61 ± 0.00	0.60 ± 0.03
PI277678	*Triticum turgidum* subsp. *dicoccon*	0.66 ± 0.03	0.56 ± 0.01	0.97 ± 0.02	0.40 ± 0.02
PI668244	*Triticum turgidum* subsp. *dicoccon*	0.65 ± 0.02	0.69 ± 0.04	0.72 ± 0.02	0.75 ± 0.02
PI668240	*Triticum turgidum* subsp. *dicoccon*	0.57 ± 0.04	0.62 ± 0.04	0.60 ± 0.01	0.65 ± 0.01
PI470780	*Triticum turgidum* subsp. *dicoccon*	0.59 ± 0.02	0.59 ± 0.02	0.84 ± 0.01	0.85 ± 0.00
PI532306	*Triticum turgidum* subsp. *dicoccon*	0.60 ± 0.00	0.62 ± 0.04	0.79 ± 0.01	0.8 ± 0.02
PI384332	*Triticum turgidum* subsp. *dicoccon*	0.65 ± 0.00	0.46 ± 0.00	0.79 ± 0.02	0.34 ± 0.02
PI308879	*Triticum turgidum* subsp. *dicoccon*	0.56 ± 0.01	0.51 ± 0.08	0.77 ± 0.02	0.7 ± 0.01
PI94613	*Triticum turgidum* subsp. *dicoccon*	0.61 ± 0.01	0.35 ± 0.00	0.67 ± 0.01	0.26 ± 0.03
PI272600	*Triticum turgidum* subsp. *dicoccon*	0.73 ± 0.02	0.45 ± 0.02	0.77 ± 0.00	0.42 ± 0.01
PI532304	*Triticum turgidum* subsp. *dicoccon*	0.69 ± 0.01	0.34 ± 0.03	0.82 ± 0.01	0.39 ± 0.03
PI470801	*Triticum turgidum* subsp. *dicoccon*	0.61 ± 0.01	0.49 ± 0.04	0.57 ± 0.01	0.17 ± 0.01
PI377672	*Triticum turgidum* subsp. *dicoccon*	0.58 ± 0.05	0.44 ± 0.02	0.77 ± 0.01	0.40 ± 0.03
PI254191	*Triticum turgidum* subsp. *dicoccon*	0.54 ± 0.01	0.43 ± 0.03	0.72 ± 0.02	0.44 ± 0.01
PI480462	*Triticum turgidum* subsp. *dicoccon*	0.62 ± 0.02	0.60 ± 0.03	0.48 ± 0.01	0.52 ± 0.02
PI480457	*Triticum turgidum* subsp. *dicoccon*	0.57 ± 0.06	0.45 ± 0.01	0.63 ± 0.01	0.57 ± 0.01
PI480312	*Triticum turgidum* subsp. *dicoccon*	0.59 ± 0.00	0.60 ± 0.01	0.45 ± 0.02	0.48 ± 0.01
PI79899	*Triticum turgidum* subsp. *dicoccon*	0.60 ± 0.03	0.26 ± 0.00	0.83 ± 0.02	0.29 ± 0.03
PI572858	*Triticum turgidum* subsp. *dicoccon*	0.67 ± 0.05	0.58 ± 0.05	0.78 ± 0.02	0.48 ± 0.00
PI374685	*Triticum turgidum* subsp. *dicoccon*	0.60 ± 0.04	0.45 ± 0.01	0.81 ± 0.02	0.38 ± 0.00
PI362696	*Triticum turgidum* subsp. *dicoccon*	0.57 ± 0.03	0.57 ± 0.01	0.80 ± 0.01	0.68 ± 0.01
PI362501	*Triticum turgidum* subsp. *dicoccon*	0.43 ± 0.03	0.45 ± 0.00	0.59 ± 0.01	0.64 ± 0.00
PI290517	*Triticum turgidum* subsp. *dicoccon*	0.48 ± 0.01	0.55 ± 0.05	0.68 ± 0.10	0.58 ± 0.05
PI182743	*Triticum turgidum* subsp. *dicoccon*	0.52 ± 0.04	0.47 ± 0.03	0.55 ± 0.02	0.54 ± 0.03
PI94640	*Triticum turgidum* subsp. *dicoccon*	0.53 ± 0.05	0.48 ± 0.00	0.40 ± 0.01	0.42 ± 0.02
PI190927	*Triticum turgidum* subsp. *dicoccon*	0.71 ± 0.08	0.48 ± 0.05	0.96 ± 0.04	0.29 ± 0.01
PI499973	*Triticum turgidum* subsp. *dicoccon*	0.58 ± 0.03	0.49 ± 0.04	0.75 ± 0.01	0.55 ± 0.02
PI480461	*Triticum turgidum* subsp. *dicoccon*	0.49 ± 0.02	0.48 ± 0.05	0.66 ± 0.02	0.70 ± 0.02
PI480068	*Triticum turgidum* subsp. *dicoccon*	0.45 ± 0.02	0.37 ± 0.02	0.65 ± 0.01	0.68 ± 0.01
PI479964	*Triticum turgidum* subsp. *dicoccon*	0.40 ± 0.00	0.42 ± 0.01	0.30 ± 0.01	0.54 ± 0.00
PI384484	*Triticum turgidum* subsp. *dicoccon*	0.39 ± 0.04	0.51 ± 0.03	0.16 ± 0.02	0.72 ± 0.00
PI341801	*Triticum turgidum* subsp. *dicoccon*	0.47 ± 0.05	0.39 ± 0.01	0.55 ± 0.02	0.40 ± 0.02
PI434999	*Triticum turgidum* subsp. *dicoccon*	0.43 ± 0.03	0.48 ± 0.02	0.51 ± 0.00	0.49 ± 0.01
PI434998	*Triticum turgidum* subsp. *dicoccon*	0.44 ± 0.02	0.43 ± 0.03	0.56 ± 0.01	0.45 ± 0.01
PI434992	*Triticum turgidum* subsp. *dicoccon*	0.44 ± 0.02	0.38 ± 0.01	0.55 ± 0.00	0.55 ± 0.00
PI377650	*Triticum turgidum* subsp. *dicoccon*	0.61 ± 0.06	0.62 ± 0.02	0.60 ± 0.01	0.54 ± 0.01
PI352337	*Triticum turgidum* subsp. *dicoccon*	0.31 ± 0.01	0.32 ± 0.03	0.40 ± 0.01	0.45 ± 0.01
PI352358	*Triticum turgidum* subsp. *dicoccon*	0.40 ± 0.01	0.44 ± 0.05	0.36 ± 0.01	0.42 ± 0.02
PI534275	*Triticum turgidum* subsp. *dicoccon*	0.53 ± 0.01	0.61 ± 0.02	0.53 ± 0.02	0.55 ± 0.02
PI191387	*Triticum turgidum* subsp. *dicoccon*	0.42 ± 0.04	0.48 ± 0.04	0.35 ± 0.01	0.48 ± 0.00
PI387685	*Triticum turgidum* subsp. *dicoccon*	0.40 ± 0.04	0.40 ± 0.05	0.37 ± 0.01	0.38 ± 0.01
PI387777	*Triticum turgidum* subsp. *dicoccon*	0.45 ± 0.01	0.52 ± 0.03	0.48 ± 0.02	0.49 ± 0.01
PI387773	*Triticum turgidum* subsp. *dicoccon*	0.42 ± 0.05	0.42 ± 0.04	0.45 ± 0.04	0.44 ± 0.01
PI387767	*Triticum turgidum* subsp. *dicoccon*	0.38 ± 0.06	0.38 ± 0.04	0.49 ± 0.01	0.51 ± 0.01
PI387750	*Triticum turgidum* subsp. *dicoccon*	0.43 ± 0.00	0.55 ± 0.01	0.38 ± 0.01	0.38 ± 0.01
PI387748	*Triticum turgidum* subsp. *dicoccon*	0.46 ± 0.04	0.40 ± 0.00	0.39 ± 0.01	0.3 ± 0.01
PI387746	*Triticum turgidum* subsp. *dicoccon*	0.45 ± 0.02	0.45 ± 0.02	0.45 ± 0.01	0.41 ± 0.01
PI387793	*Triticum turgidum* subsp. *dicoccon*	0.27 ± 0.00	0.41 ± 0.02	0.26 ± 0.01	0.26 ± 0.03
PI387792	*Triticum turgidum* subsp. *dicoccon*	0.46 ± 0.04	0.34 ± 0.00	0.30 ± 0.02	0.08 ± 0.00
PI94624	*Triticum turgidum* subsp. *dicoccon*	0.47 ± 0.05	0.53 ± 0.00	0.58 ± 0.01	0.61 ± 0.01
CItr14085	*Triticum turgidum* subsp. *dicoccon*	0.55 ± 0.02	0.58 ± 0.00	0.80 ± 0.01	0.41 ± 0.02
PI94625	*Triticum turgidum* subsp. *dicoccon*	0.61 ± 0.03	0.60 ± 0.03	0.68 ± 0.01	0.73 ± 0.01
PI190923	*Triticum turgidum* subsp. *dicoccon*	0.30 ± 0.02	0.44 ± 0.02	0.12 ± 0.01	0.24 ± 0.01
PI326312	*Triticum turgidum* subsp. *dicoccon*	0.50 ± 0.07	0.36 ± 0.00	0.71 ± 0.01	0.04 ± 0.00
PI330544	*Triticum turgidum* subsp. *dicoccon*	0.44 ± 0.01	0.42 ± 0.05	0.37 ± 0.01	0.34 ± 0.01
PI352369	*Triticum turgidum* subsp. *dicoccon*	0.38 ± 0.01	0.38 ± 0.00	0.34 ± 0.01	0.46 ± 0.01
PI384305	*Triticum turgidum* subsp. *dicoccon*	0.39 ± 0.01	0.40 ± 0.01	0.33 ± 0.01	0.32 ± 0.01
CItr14838	*Triticum turgidum* subsp. *dicoccon*	0.30 ± 0.03	0.43 ± 0.01	0.56 ± 0.02	0.11 ± 0.00
CItr14637	*Triticum turgidum* subsp. *dicoccon*	0.46 ± 0.03	0.44 ± 0.04	0.84 ± 0.01	0.38 ± 0.01
PI94631	*Triticum turgidum* subsp. *dicoccon*	0.36 ± 0.02	0.34 ± 0.00	0.26 ± 0.01	0.29 ± 0.01
PI94630	*Triticum turgidum* subsp. *dicoccon*	0.52 ± 0.02	0.35 ± 0.02	0.80 ± 0.01	0.07 ± 0.00
CItr14972	*Triticum turgidum* subsp. *dicoccon*	0.51 ± 0.04	0.43 ± 0.02	0.57 ± 0.00	0.41 ± 0.01
CItr14971	*Triticum turgidum* subsp. *dicoccon*	0.50 ± 0.02	0.39 ± 0.01	0.51 ± 0.00	0.26 ± 0.01
CItr14917	*Triticum turgidum* subsp. *dicoccon*	0.36 ± 0.01	0.44 ± 0.00	0.12 ± 0.01	0.50 ± 0.01
CItr14868	*Triticum turgidum* subsp. *dicoccon*	0.38 ± 0.01	0.33 ± 0.03	0.30 ± 0.01	0.30 ± 0.01
CItr14867	*Triticum turgidum* subsp. *dicoccon*	0.58 ± 0.03	0.40 ± 0.01	0.52 ± 0.02	0.23 ± 0.01
CItr14866	*Triticum turgidum* subsp. *dicoccon*	0.53 ± 0.02	0.54 ± 0.02	0.37 ± 0.01	0.39 ± 0.01
PI94662	*Triticum turgidum* subsp. *dicoccon*	0.50 ± 0.03	0.46 ± 0.05	0.55 ± 0.01	0.36 ± 0.01
PI94661	*Triticum turgidum* subsp. *dicoccon*	0.50 ± 0.05	0.34 ± 0.02	0.63 ± 0.01	0.12 ± 0.01
PI94648	*Triticum turgidum* subsp. *dicoccon*	0.40 ± 0.03	0.53 ± 0.03	0.76 ± 0.02	0.27 ± 0.01
PI94636	*Triticum turgidum* subsp. *dicoccon*	0.62 ± 0.04	0.26 ± 0.01	0.74 ± 0.00	0.17 ± 0.01
PI164578	*Triticum turgidum* subsp. *dicoccon*	0.56 ± 0.07	0.46 ± 0.01	0.73 ± 0.01	0.06 ± 0.00
PI94682	*Triticum turgidum* subsp. *dicoccon*	0.55 ± 0.07	0.34 ± 0.01	0.84 ± 0.02	0.05 ± 0.01
PI94681	*Triticum turgidum* subsp. *dicoccon*	0.48 ± 0.04	0.36 ± 0.02	0.56 ± 0.02	0.06 ± 0.00
PI94675	*Triticum turgidum* subsp. *dicoccon*	0.47 ± 0.01	0.35 ± 0.02	0.75 ± 0.01	0.40 ± 0.00
PI193878	*Triticum turgidum* subsp. *dicoccon*	0.54 ± 0.03	0.37 ± 0.03	0.64 ± 0.02	0.31 ± 0.00
PI193644	*Triticum turgidum* subsp. *dicoccon*	0.45 ± 0.04	0.51 ± 0.00	0.47 ± 0.01	0.49 ± 0.01
PI193643	*Triticum turgidum* subsp. *dicoccon*	0.38 ± 0.00	0.38 ± 0.03	0.38 ± 0.01	0.38 ± 0.01
PI193642	*Triticum turgidum* subsp. *dicoccon*	0.63 ± 0.03	0.33 ± 0.00	0.82 ± 0.01	0.06 ± 0.00
PI195722	*Triticum turgidum* subsp. *dicoccon*	0.59 ± 0.04	0.31 ± 0.02	0.86 ± 0.01	0.24 ± 0.02
PI194375	*Triticum turgidum* subsp. *dicoccon*	0.45 ± 0.04	0.43 ± 0.02	0.72 ± 0.01	0.70 ± 0.01
PI254150	*Triticum turgidum* subsp. *dicoccon*	0.37 ± 0.00	0.4 ± 0.00	0.57 ± 0.02	0.49 ± 0.01
PI234868	*Triticum turgidum* subsp. *dicoccon*	0.46 ± 0.00	0.29 ± 0.03	0.60 ± 0.02	0.44 ± 0.01
PI221401	*Triticum turgidum* subsp. *dicoccon*	0.50 ± 0.07	0.50 ± 0.04	0.88 ± 0.01	0.78 ± 0.01
PI221400	*Triticum turgidum* subsp. *dicoccon*	0.37 ± 0.03	0.42 ± 0.01	0.71 ± 0.01	0.79 ± 0.01
PI197495	*Triticum turgidum* subsp. *dicoccon*	0.71 ± 0.06	0.34 ± 0.02	0.79 ± 0.02	0.33 ± 0.01
PI254173	*Triticum turgidum* subsp. *dicoccon*	0.63 ± 0.00	0.33 ± 0.03	0.82 ± 0.01	0.19 ± 0.01
PI254169	*Triticum turgidum* subsp. *dicoccon*	0.61 ± 0.06	0.42 ± 0.00	0.56 ± 0.02	0.35 ± 0.02
PI254168	*Triticum turgidum* subsp. *dicoccon*	0.49 ± 0.00	0.41 ± 0.01	0.63 ± 0.01	0.62 ± 0.01
PI254160	*Triticum turgidum* subsp. *dicoccon*	0.36 ± 0.01	0.42 ± 0.01	0.38 ± 0.01	0.38 ± 0.02
PI254152	*Triticum turgidum* subsp. *dicoccon*	0.42 ± 0.05	0.37 ± 0.01	0.36 ± 0.01	0.35 ± 0.01
PI254186	*Triticum turgidum* subsp. *dicoccon*	0.41 ± 0.02	0.41 ± 0.05	0.26 ± 0.01	0.16 ± 0.01
PI254182	*Triticum turgidum* subsp. *dicoccon*	0.31 ± 0.03	0.32 ± 0.01	0.27 ± 0.00	0.51 ± 0.01
PI254179	*Triticum turgidum* subsp. *dicoccon*	0.52 ± 0.02	0.50 ± 0.03	0.49 ± 0.03	0.18 ± 0.00
PI254178	*Triticum turgidum* subsp. *dicoccon*	0.47 ± 0.04	0.47 ± 0.01	0.73 ± 0.01	0.51 ± 0.02
PI254177	*Triticum turgidum* subsp. *dicoccon*	0.45 ± 0.05	0.35 ± 0.00	0.65 ± 0.01	0.37 ± 0.00
PI275996	*Triticum turgidum* subsp. *dicoccon*	0.39 ± 0.04	0.44 ± 0.03	0.42 ± 0.00	0.44 ± 0.00
PI273982	*Triticum turgidum* subsp. *dicoccon*	0.35 ± 0.02	0.53 ± 0.00	0.09 ± 0.02	0.39 ± 0.00
PI276020	*Triticum turgidum* subsp. *dicoccon*	0.33 ± 0.01	0.45 ± 0.05	0.44 ± 0.02	0.42 ± 0.01
PI276017	*Triticum turgidum* subsp. *dicoccon*	0.42 ± 0.01	0.41 ± 0.02	0.44 ± 0.07	0.42 ± 0.02
PI276008	*Triticum turgidum* subsp. *dicoccon*	0.44 ± 0.04	0.44 ± 0.01	0.42 ± 0.01	0.34 ± 0.02
PI276003	*Triticum turgidum* subsp. *dicoccon*	0.47 ± 0.04	0.41 ± 0.04	0.92 ± 0.03	0.37 ± 0.00
PI298573	*Triticum turgidum* subsp. *dicoccon*	0.32 ± 0.01	0.52 ± 0.05	0.23 ± 0.00	0.22 ± 0.00
PI322232	*Triticum turgidum* subsp. *dicoccon*	0.35 ± 0.01	0.41 ± 0.05	0.34 ± 0.00	0.49 ± 0.01
PI319868	*Triticum turgidum* subsp. *dicoccon*	0.58 ± 0.01	0.42 ± 0.04	0.48 ± 0.02	0.43 ± 0.00
PI310471	*Triticum turgidum* subsp. *dicoccon*	0.48 ± 0.01	0.31 ± 0.00	0.52 ± 0.06	0.40 ± 0.02
PI306538	*Triticum turgidum* subsp. *dicoccon*	0.39 ± 0.02	0.52 ± 0.01	0.56 ± 0.00	0.74 ± 0.02
PI306537	*Triticum turgidum* subsp. *dicoccon*	0.67 ± 0.04	0.55 ± 0.05	0.82 ± 0.04	0.48 ± 0.01
PI352350	*Triticum turgidum* subsp. *dicoccon*	0.32 ± 0.02	0.44 ± 0.02	0.45 ± 0.01	0.53 ± 0.02
PI355476	*Triticum turgidum* subsp. *dicoccon*	0.55 ± 0.01	0.52 ± 0.04	0.38 ± 0.00	0.63 ± 0.01
PI355469	*Triticum turgidum* subsp. *dicoccon*	0.33 ± 0.02	0.54 ± 0.04	0.61 ± 0.01	0.16 ± 0.01
PI355467	*Triticum turgidum* subsp. *dicoccon*	0.51 ± 0.02	0.45 ± 0.01	0.61 ± 0.01	0.28 ± 0.01
PI355464	*Triticum turgidum* subsp. *dicoccon*	0.69 ± 0.04	0.33 ± 0.01	0.91 ± 0.01	0.37 ± 0.01
PI355460	*Triticum turgidum* subsp. *dicoccon*	0.45 ± 0.04	0.44 ± 0.04	0.68 ± 0.00	0.83 ± 0.01
PI384301	*Triticum turgidum* subsp. *dicoccon*	0.50 ± 0.01	0.40 ± 0.00	0.85 ± 0.01	0.59 ± 0.00
PI377660	*Triticum turgidum* subsp. *dicoccon*	0.50 ± 0.00	0.55 ± 0.01	0.88 ± 0.01	0.63 ± 0.05
PI377658	*Triticum turgidum* subsp. *dicoccon*	0.46 ± 0.01	0.46 ± 0.00	0.57 ± 0.00	0.56 ± 0.00
PI377657	*Triticum turgidum* subsp. *dicoccon*	0.51 ± 0.01	0.53 ± 0.05	0.59 ± 0.04	0.55 ± 0.01
PI377655	*Triticum turgidum* subsp. *dicoccon*	0.54 ± 0.06	0.52 ± 0.02	0.76 ± 0.01	0.82 ± 0.02
PI384320	*Triticum turgidum* subsp. *dicoccon*	0.41 ± 0.05	0.44 ± 0.04	0.47 ± 0.02	0.48 ± 0.02
PI387699	*Triticum turgidum* subsp. *dicoccon*	0.45 ± 0.00	0.50 ± 0.01	0.74 ± 0.01	0.76 ± 0.01
PI387683	*Triticum turgidum* subsp. *dicoccon*	0.69 ± 0.05	0.54 ± 0.06	0.75 ± 0.01	0.65 ± 0.00
PI418586	*Triticum turgidum* subsp. *paleocolchicum*	0.77 ± 0.04	0.48 ± 0.00	0.75 ± 0.01	0.57 ± 0.01
PI349050	*Triticum turgidum* subsp. *paleocolchicum*	0.52 ± 0.00	0.41 ± 0.00	0.60 ± 0.01	0.44 ± 0.02
PI192666	*Triticum turgidum* subsp. *polonicum*	0.49 ± 0.00	0.31 ± 0.02	0.77 ± 0.01	0.52 ± 0.00
CItr14803	*Triticum turgidum* subsp. *polonicum*	0.52 ± 0.05	0.44 ± 0.00	0.75 ± 0.02	0.07 ± 0.01
PI387457	*Triticum turgidum* subsp. *polonicum*	0.63 ± 0.03	0.59 ± 0.06	0.80 ± 0.03	0.35 ± 0.01
PI290512	*Triticum turgidum* subsp. *polonicum*	0.73 ± 0.04	0.37 ± 0.01	0.69 ± 0.01	0.37 ± 0.01
PI272566	*Triticum turgidum* subsp. *polonicum*	0.42 ± 0.00	0.53 ± 0.02	0.73 ± 0.01	0.76 ± 0.03
PI225334	*Triticum turgidum* subsp. *polonicum*	0.51 ± 0.01	0.49 ± 0.03	0.56 ± 0.02	0.21 ± 0.01
PI384343	*Triticum turgidum* subsp. *polonicum*	0.34 ± 0.01	0.51 ± 0.01	0.41 ± 0.01	0.69 ± 0.02
PI566593	*Triticum turgidum* subsp. *polonicum*	0.47 ± 0.04	0.40 ± 0.01	0.42 ± 0.01	0.41 ± 0.00
CItr14095	*Triticum turgidum* subsp. *turanicum*	0.35 ± 0.00	0.52 ± 0.03	0.28 ± 0.00	0.42 ± 0.01
PI190973	*Triticum turgidum* subsp. *turanicum*	0.48 ± 0.04	0.45 ± 0.01	0.85 ± 0.00	0.38 ± 0.01
PI306665	*Triticum turgidum* subsp. *turanicum*	0.48 ± 0.04	0.45 ± 0.01	0.34 ± 0.02	0.32 ± 0.01
PI166554	*Triticum turgidum* subsp. *turanicum*	0.49 ± 0.02	0.43 ± 0.06	0.70 ± 0.02	0.70 ± 0.02
PI254205	*Triticum turgidum* subsp. *turanicum*	0.64 ± 0.02	-	0.55 ± 0.00	-
PI211691	*Triticum turgidum* subsp. *turanicum*	0.48 ± 0.04	0.35 ± 0.01	0.45 ± 0.01	0.06 ± 0.00
PI166450	*Triticum turgidum* subsp. *turanicum*	0.36 ± 0.03	0.39 ± 0.03	0.27 ± 0.02	0.28 ± 0.00
PI256034	*Triticum turgidum* subsp. *turanicum*	0.60 ± 0.05	0.44 ± 0.02	0.6 ± 0.00	0.17 ± 0.00
PI272602	*Triticum turgidum* subsp. *turanicum*	0.44 ± 0.02	0.41 ± 0.01	0.46 ± 0.00	0.27 ± 0.01

GAE = gallic acid equivalent; TE = Trolox equivalent. Data are presented as means ± SD of replicates.

**Table 2:** Descriptive statistics for 144 tetraploid wheat genotypes for total phenolic content (TPC) and antioxidant activity (AA), averaged per environment.

Trait	Statistics	Chile	Italy
TPC	Mean	0.50	0.45
	SD (±)	0.02	0.02
	Median	0.49	0.44
	Min	0.27	0.26
	Max	0.82	0.79
	*H* ^2^	0.72	
AA	Mean	0.58	0.44
	SD (±)	0.01	0.01
	Median	0.59	0.42
	Min	0.08	0.04
	Max	0.99	0.89
	*H* ^2^	0.65	

**Table 3:** Analysis of variance (ANOVA) for total phenolic content (TPC) and antioxidant activity (AA) in 144 wheat tetraploid accessions grown in Chile and in Italy.

Parameter	Environment	Source	df	MS	F Value	
TPC	Chile	Replication	1	0.0017014	1.550	ns
		Genotype	143	0.0240493	21.905	***
	Italy	Replication	1	0.0039185	5.094	*
		Genotype	142	0.0164323	21.364	***
		Genotype x environment	142	0.0150100	15.840	***
AA	Chile	Replication	1	0.003042	9.614	**
		Genotype	143	0.076817	242.794	***
	Italy	Replication	1	0.000253	1.314	ns
		Genotype	142	0.076967	399.484	***
		Genotype x environment	142	0.065100	245.200	***

*** *p* ≤ 0.001, ** *p* ≤ 0.01, * *p* ≤ 0.05, ns = not significant.

**Table 4:** SNP markers significantly associated (LOD ≥ 3) with total phenolic content (TPC, expressed as gallic acid equivalent, GAE) and antioxidant activity (AA, expressed as Trolox equivalent, TE) identified by GWAS in the whole tetraploid wheat collection evaluated in two environments (Chile and Italy) and the associated genes.

Traits	QTL	Closest Marker	Marker ID	SNP Alleles	Chr	cM	Position (bp)	Environments						Mean			**Candidate Gene**
								Chile			Italy						
								LOD	R^2^	Marker Effect	LOD	R^2^	Marker Effect	LOD	R^2^	Marker Effect	
TPC	*QGae.bc.2B*	AX_158547367			2B		30,497,225	-	-	-	-	-	-	3.30	0.10	0.02	
	*QGae.bc.3A*	AX_158523192			3A		617,303,886	-	-	-	3.10	0.09	−0.05	-	-	-	flavonol 3-sulfotransferase
	*QGae.bc.4B*	AX_158556017			4B		37,431,007	-	-	-	-	-	-	5.30	0.16	−0.03	
	*QGae.bc.5A*	BS00065481_51	IWB9564	T/C	5A	141.3	581,479,161	4.83	0.14	0.46	-	-	-	-	-	-	
AA	*QTe.bc.2A-1*	AX_158573306			2A		62,235,644	-	-	-	-	-	-	3.05	0.09	0.06	
	*QTe.bc.2A-2*	AX_110949499			2A		694,583,696	-	-	-	-	-	-	3.65	0.11	−0.05	
	*QTe.bc.2A-3*	AX_158540693			2A		715,414,711	5.73	0.17	−0.01	-	-	-	-	-	-	
	*QTe.bc.2B-1*	Excalibur_c31042_178	IWB25055	T/C	2B	12.3	17,390,640	3.37	0.10	0.11	-	-	-	-	-	-	
	*QTe.bc.2B-2*	AX_158575044			2B		45,139,675	3.41	0.10	−0.11	-	-	-	-	-	-	
	*QTe.bc.4B*	AX_158556017			4B		37,431,007	-	-	-	-	-	-	3.18	0.10	−0.04	
	*QTe.bc.5A-1*	wsnp_Ku_c14275_22535576	IWA6522	T/C	5A	90.3	478,821,006	5.73	0.17	−0.06	-	-	-	-	-	-	
	*QTe.bc.5A-2*	BS00065481_51	IWB9564	T/C	5A	141.3	581,479,161	5.73	0.17	0.58	-	-	-	-	-	-	
	*QTe.bc.5B-1*	AX_94534815			5B		24,114,456	3.11	0.09	0.08	-	-	-	-	-	-	
	*QTe.bc.5B-2*	BS00068805_51	IWB10362	A/C	5B	113.9	551,499,561	-	-	-	3.16	0.10	0.07	3.40	0.10	0.06	
	*QTe.bc.6A*	AX_94494977			6A		597,938,935	5.73	0.17	−0.08	-	-	-	-	-	-	Peptidylprolyl isomerase
	*QTe.bc.6B-1*	AX_109495285			6B		55,647,740	3.73	0.11	0.14	-	-	-	-	-	-	
	*QTe.bc.6B-2*	AX_109345149			6B		123,748,576	5.73	0.17	−0.05	-	-	-	-	-	-	

## Supplementary Materials

The following supporting information can be downloaded at: https://www.mdpi.com/article/10.3390/antiox14091048/s1, Table S1: Meterological data from Pirque experimental station, Italy. Table S2: Meterological data from Pirque experimental station, Chile.
